# Observations on Fever

**Published:** 1828-03

**Authors:** 

**Affiliations:** Alnwick.


### THE LONDON
### Medical and Physical Journal.

N° 349, VOL. LIX.] MARCH, 1828. [N° 21, *New Series.*

**For many fortunate discoveries in medicine, and for the detection of numerous errors, the world is indebted to the rapid circulation of Monthly Journals; and there never existed any work, to which the Faculty, in Europe and America, were under deeper obligations, than to the Medical and Physical Journal of London, now forming a long, but an invaluable, series.—RUSH.**

### ORIGINAL PAPERS,

AND

### CASES OBTAINED FROM PUBLIC INSTITUTIONS AND OTHER AUTHENTIC SOURCES.

### FEVER.

*Observations on Fever.* Addressed to a Medical Friend, by Dr. Bow, of Alnwick.

ACCORDING to promise, my subject must be the theory of fever, although it is one I would willingly decline; for I now think I was presumptuous when I proposed to enter a field where so many able men have toiled. Nevertheless, the sources of those symptoms to which the term fever is attached are still concealed; and, since profound research, skill, and ingenuity have failed, chance may direct the steps of the humblest labourer, or a lucky thought may give the clue by which the mystery may be unravelled: this can alone support an adventurer such as I am, and be his only apology for venturing to meddle with this most difficult of all speculative subjects.

You will agree with me that a theory of fever is wanted: one, at least, which might in some degree guide the practitioner; for even now, notwithstanding the many that have seen the light, we are forced to acknowledge that we grope in the dark, and to confess that our practice owes its success more to chance than to science. I know it to be the opinion of some that a rational and successful plan of treatment may be found independent of the knowledge of the proximate cause of fever. In this opinion, however, I cannot coincide; for, although we be successful in our practice, and therefore call our plan rational, we but combat a symptom as it appears with the remedies with which experience has armed us, and we anticipate another which experience teaches us to expect; but we know not what gives birth to the one, nor how the action we induce operates in preventing the other. A knowledge of the origin of the phenomena of fever cannot be prejudicial to the practitioner; neither can an attempt to investigate it render him who makes it less skilful in his art. Perhaps our physiological knowledge has not arrived at that perfection which might authorise us to pronounce upon the functional derangements which give rise to symptoms, yet I think careful observation, induction from physiological facts, a proper application of pathological research and experiment, and a judicious culling from the theories extant, may yield principles approaching to truth, and convey to us some idea of the nature of fever. CULLEN put so much value on the knowledge of the proximate causes of diseases, that he believed the cure to be founded chiefly, and almost unavoidably, on that knowledge; and it was with a view to the better conducting of the practice in fevers that he reared his theory, a structure which, as Dr. GOOD remarks, "if it be at this moment crumbling into decay, certainly is not falling prostrate before any fabric of more substantial materials, or more elegant architecture."

Every unbiassed physician believes with Cullen that the phenomena of fever in its earliest stage depend on some remote cause acting immediately on the nervous system; and, if the symptoms themselves did not point to this origin, the very sudden invasion of fevers, in many cases, is sufficient to warrant the conclusion.

The remote causes are certain sedative powers which diminish the energy of the brain. Though there be yet much to learn regarding the functions of the brain, I think, if we pursue our inquiry with the knowledge we do possess, we may arrive at something satisfactory. For the sake of distinctness, let me explain what I understand by the energy of the brain, or rather in what sense I wish to be understood. By energy, I understand activity, a mere quality. By nervous influence, I mean the nervous fluid secreted by the brain and spinal marrow, and emanating from them. It appears that there are modifications of this influence, flowing by separate and distinct nerves; or, if not, the nerves themselves are so modified that, carrying the same matter, they perform separate and distinct functions. Such a modification was at first suspected, and is now proved by experiment; one set of nerves producing the effect of sensation, another set the effect of motion. But there is another modification, it is strongly suspected, either of nerve or nervous influence, which is supposed to play an important part in the animal economy, which, were it substantiated by experiment or otherwise, would, I think, enable us to thread the pathology of fever; and, in my opinion, the arguments in favor of such a modification are of more weight than those opposed to it. It is that modification of nerve or nervous influence which is supposed to effect secretion. The nervous hypothesis of secretion appears to me to be the only plausible explanation of this operation,—the only one which can be fairly and legitimately adopted; and, indeed, the objections brought forward by its opponents are attempts made rather to weaken the inferences drawn in its support, than facts directly opposed to it. Having said thus much, I think it incumbent on me to advert to some of those objections, and I trust you will pardon the digression, not only because secretion is a subject in itself highly interesting, but because, unless we can form some idea of the real nature of the function, we may bid adieu to the hope of tracing the proximate causes of idiopathic diseases.

Dr. Good concludes, with Sir E. Home, that "the organs of secretion are principally made up of arteries and veins, but there is nothing in the different modes in which these vessels ramify that can in any way account for the changes in the blood, out of which secretions arise." These organs being largely supplied with twigs of small nerves, naturally gave rise to the idea that through their instrumentality is secretion effected. In support of this view, Sir E. Home observes, "that, in fishes which are capable of secreting the electrical fluid, the nerves connected with the electrical organs exceed those that go to all the other parts of the fish, in the proportion of twenty to one; and, in confirmation of this view of the subject, Dr. Good remarks, "that there are no parts of the body more manifestly affected, and few so much so, as the secretory organs, by mental emotions. "The whole surface of the skin is sometimes bedewed with drops of sweat, and even of blood, by a sudden paroxysm of agony of mind; grief fills the eyes with tears; fear is well known to be a powerful stimulant to the kidneys, and very generally to the alvine canal; anger gives an additional flow, perhaps an additional acrimony, to the bile, and, if urged to violence, renders the saliva of some animals poisonous; and disappointed hope destroys the digestion, and turns the secreted fluids of the stomach acid." Although the above seems to prove that the secretory organs are chiefly influenced by the sensorial system, yet it is denied that secretion is effected by nervous influence, because HALLER observed that the larger branches of the nerves seldom enter into the secretory organs, and seem purposely to avoid them; because the secernent glands have little sensibility; and because the secretions of plants, which have no nervous system, are as abundant and diversified, and as wonderful in every respect, as those of animals. In all this I do not see one valid objection: it is now known that there are two distinct classes of nerves, independent of the great sympathetic,—the motor nerves, which can have no necessary connexion with the secernent glands, do avoid them, and even that perhaps purposely; and, if the glands possess but little sensibility, although largely supplied with twigs of small nerves, it is evident that all these twigs cannot proceed from sentient nerves, and, as no other nerves are acknowledged but those arising from the brain, spinal marrow, and great sympathetic, it is evident they must proceed from the last mentioned; the more especially as branches from the great sympathetic can be traced to every gland and every secreting surface.

Plants are living structures to be sure, but they differ from animals, inasmuch as they exhibit neither signs of sensation nor of voluntary motion: consequently we do not expect to find in them either nerves of sensation or of volition. They differ in another point of view, which ought to lead us to think that the operations in the vegetable kingdom are principally effected by external influence: the laboratory of the vegetable kingdom is external, whilst that of the animal kingdom is internal. In this respect, plants are indeed the inverted animals of Dr. ALSTON. LINNEUS has been blamed for too fondly searching after analogies by which he might approximate the vegetable to the animal system; but it would appear that we are possessed of a similar propensity, since we reject a plausible explanation of an operation carried on in the animal, because, forsooth, we cannot analogically explain a seemingly similar operation going on in the vegetable. That the sunbeam possesses rays endowed with a deoxidizing power, the analysis of light has revealed to us. By exposure to these rays, and to atmospheric action, is the sap modified in the leaf; it is then conveyed to the cells in the parenchyma, where, by further exposure, it is fitted for the purposes required of it; and as in the light plants produce oxygen gas, when in the dark they do not, we can only conceive that oxygen is separated in consequence of some new combination effected through the agency of light. Thus light may effect the decompositions and recombinations necessary to the vegetable, whilst nervous influence performs these processes in the animal: hence vegetable secretion is influenced by locality and the seasons, animal secretion by whatever perceptibly operates on the nervous system.

Dr. Bostock's principal objection to the nervous hypothesis, and which, by the bye, he brings forward as a direct argument in opposition to it, is, that secretion appeared to be produced in fœtuses which had no nervous system. I have no opportunity of examining the cases cited, but, admitting that in all of them the brain, cerebellum, and spinal marrow were wanting, it does not follow there was no nervous system, for in similar cases the sympathetic nerves have been found perfect; and, if the great sympathetic has more than one function, I am inclined to believe that secretion in general is performed through its instrumentality. Organs so important as the brain and spinal marrow are protected from external injury by their bony casements; but the sympathetic, although it has no such covering, yet, from its situation, it is still better protected, and, as nature does nothing without an object, we might infer that its functions are highly important. And what function, the due performance of which is more necessary to health and life than secretion. Sensation or voluntary motion may be impaired or almost wholly destroyed without necessarily causing death, yet these are functions performed through the instrumentality of separate and distinct nerves. Secretion is a function which cannot even in part be destroyed without endangering life, yet we deny that this function is performed through nervous agency, although we can trace nerves proceeding to the organs of secretion, knowing at the same time that they are nerves neither of sensation, nor of voluntary nor of involuntary motion.

I have said that, by nervous influence, I mean the nervous fluid secreted by the brain and spinal marrow; but, by saying so, I do not wish to be deprived of the privilege of supposing that nervous fluid may not be secreted by the great sympathetic, and emanate from it also, for I do not think that Bichat was altogether incorrect in supposing that this nerve is independent of the great system of nerves. Anatomists do not say that the sympathetic arises from the sixth pair; for it is supposed that, instead of receiving filaments, it gives filaments to that pair. Or, according to Shaw, "when the foramen caroticum is opened, a plexus of nerves will be found surrounding the carotid artery, which appear to be united with the sixth; but which, when carefully traced, will be found to pass over the sixth, to unite with the casserian ganglion of the fifth. There will also be branches seen passing along the vidian nerve, to form in union with it the ganglion of Meckel." From repeated dissections, Mr. Shaw concludes that the fifth pair resembles the spinal nerves in every respect; and by the fact that many of the branches of the sympathetic *go to* the ganglionic portion of the fifth, it is proved, he says, that even the connexion between the sympathetic and fifth is *similar* to the union of the sympathetic with the ganglionic roots of the spinal nerves. Notwithstanding this similarity of connexion, Mr. Shaw, it will be observed, in speaking of the branches of the sympathetic which join the ganglion of the fifth, constantly says that they go to the ganglion; whereas, in speaking of the spinal nerves, he says, "we shall find that each nerve has a double root,—i. e. one from the anterior and the other from the posterior column of the spinal marrow; that the one from the posterior has, immediately before it joins with the anterior, a ganglion formed upon it; and, if we carefully examine this, we shall find that from each ganglion a small nerve is *sent off* to join with the sympathetic."* The mode in which the sympathetic proceeds to and joins the casserian ganglion evidently proves that we are correct in saying that branches pass over the sixth pair to unite with this ganglion; and consequently, in describing the branches which pass off from the casserian ganglion, Mr. Shaw takes no notice of the sympathetic; whereas, since he has proved the union of the sympathetic with the ganglionic roots of the spinal nerves to be similar to the connexion between the sympathetic and the fifth, we certainly ought to believe, from analogy alone, that the sympathetic rather gives than receives a branch from the ganglionic roots.

The idea that the sympathetic is an independent nerve is almost confirmed by the observation that it has been found perfect in monsters in which the brain and spinal marrow have been found deficient; but this, it is said, forms no argument for its independency, for in such creatures the spinal nerves are also found, which are acknowledged to have their origin from the spinal marrow.†

This, however, appears to me not only to form no argument against the sympathetic being an isolated nerve, but to form one in favor of its being instrumental in assimilation, Mr. Shaw, who opposes the inferences drawn from the effects

* Manual of Anatomy, by John Shaw.

† Shaw on the Superadded Nerves, (London Medical and Physical Journal, vol. 59.)

which follow the division of the par vagum, says that the same deductions have been drawn from experiments where the par vagum and sympathetic have been cut, as from those where only the par vagum was divided. This, however, so far from weakening the inferences drawn in regard to the par vagum, gives us cause to impute a similar power to the sympathetic,—viz. that it also is a nerve of secretion. If the stomach depended altogether on the sympathetic system of nerves for its supply of nervous influence, health, nay life itself, could be but the tenure of a moment, liable to be extinguished even by the ingesta meant to support it. But, to guard against this, the stomach has been provided with a pair of nerves derived from the brain, through which, when a supply of gastric fluid is required, is the influence necessary to its secretion transmitted. Were it not for those nerves, the demand for nervous influence must have been made on the sympathetic system, and the supply granted at the expense of other vital organs. Even as it is, when nervous energy is very low, danger constantly attends a sudden call for nervous influence. A full meal to a person reduced to the last extreme by hunger is followed by death, because, as the brain cannot immediately supply the necessary influence, the ganglionic system is drained for the purpose, and the heart ceases to pulsate: in like manner, a cathartic one reduced by disease, causes death by determining to the gut the little of influence with which the ganglionic system is then supplied. In fishes, the great sympathetic is extremely slender, but, as if to compensate for this, the eighth pair is remarkable for its size and distribution: a branch of it even extends under the skin as far as the tail-fin,* evidently for the purpose of effecting the secretion of that slimy fluid which protects the skin. In the skate, that slimy fluid issues from ducts which terminate on almost the whole surface of the skin. These ducts Dr. Monro has traced from central parts, from whence they radiate, and there a pair of nerves terminate, which, before they divide, are nearly as large as the optic.† For what purpose, then, are such large nerves sent to these ducts, if it be not to effect the secretion of their contents.

Some of the oppositionists to the nervous hypothesis not only deny that the nerves are concerned in the process of secretion, but they would have us to believe that in some instances nervous action retards and disturbs the process. Nervous action is found in some instances even to retard and disturb the assimilating process; for it is a matter of obser-

* Outlines of Comparative Anatomy, by Andrew Fife. † Ibid.

vation that, in many cases of hemiplegia, where the nervous power is withdrawn or impaired, digestion goes on better than in ordinary health, and an obstinate ulcer has been found to heal quickly after the limb was struck with palsy."*

Were I to search for facts as arguments in favor of the hypothesis, I should certainly draw upon the above, and infer that digestion depends upon nervous inasmuch as, in many cases of hemiplegia, it goes better on than in ordinary health. We have no proof that, in many such cases, nervous influence is not secreted : we know, however, that it is not transmitted as usual to the paralytic members, and therefore it is but reasonable to suppose that other parts of the body will be better supplied. Upon the same principle, opiate frictions improve digestion, and accelerate the healing of ulcers.

As there are at least grounds for supposing that secretion is a nervous process, we cannot help forming to ourselves some idea of the nature of that process; and, if our ideas do not actually militate against any known law of the system, there can be no harm in arranging them even on assumption.

Let us suppose the blood to be acted on by the nervous influence, after the manner in which fluids are acted on by galvanism, and we can conceive no apparatus more exquisitely fitted for the purpose of secretion than a gland. In a gland, the arteries soon become so minute that they cannot carry forward the grosser particles of the blood : these are returned by the veins, whilst the finer stream is carried towards the secretory duct; but in its passage there, and in its most extenuated state, it is subjected to the decomposing power of the numerous small nerves which proceed from the sympathetic; its elementary affinity is subverted, and the minuteness of the vessels which favored the decomposition favors likewise the recombination; and, as the absence or presence of one atom is only necessary to change in toto the appearance and properties of matter, so we have, according to the force of the decomposing power, and the circumstances under which the recombination takes place, a diversity of fluids, differing more or less from the fluid furnishing the elements.

Yet, after all, the admission on all sides that the nerves possess an influence over the secretory organs, is sufficient for my purpose. The remote causes of fever being certain sedative powers which diminish the energy of the brain and nervous system, let us inquire what we ought to expect from such diminished energy. As the general condition both of

* Elements of Medical Logick, by Sir G. Blane, Bart.

body and mind depends upon the operative state of the brain, these must become enfeebled in all their powers, as a natural consequence of diminished energy of that organ. This enfeebled state is manifested by listlessness, dejection of spirits, and muscular debility.

But there are other symptoms, which our knowledge of the functions of the nervous system enables us to trace to the same cause. We know that the capillary arteries are under the influence of the brain, that they forward their contents by a power regulated by it; and the knowledge of this fact explains to us why paleness of the face, and cold sensations on the surface of the body, are invariably among the primary symptoms of fever. For, owing to the want of power in the vessels to carry on the circulation, the blood forsakes the surface: hence, were nervous influence present sufficient even for the evolution of heat, one material necessary to its production is in a great measure wanting. The diminished energy of the brain is sufficient in itself to account for the low quick pulse, but, as the heart is also influenced by its contents, we may there detect an additional cause of this symptom.

The change which the blood undergoes in the lungs is altogether depending on the nervous influence: if this influence, then, be not supplied in due force, that change cannot be duly effected, and the heart can only receive blood wanting in its stimulating principle. As all nerves require a regular supply of influence in order to the due fulfilment of their functions, all must be affected more or less when the energy of the brain becomes impaired; therefore the nerves supplying the respiratory apparatus share the effects. The diaphragm cannot contract as it ought to do, and the muscles destined to raise the ribs are unfitted for their office: hence the sense of weight or anxiety about the precordia, occasional sighing, or hurried breathing.

The particular sensation of cold of the back and pain of the loins may be referred to venous congestion, caused by the retrocession of blood from the surface pressing on the dorsal and lumbar twigs as they emerge from the spinal canal; for we know that pressure or irritation of a nerve in its course may give rise to unpleasant or painful sensations at its extremity, while the same pressure on the motor twigs must undoubtedly be the cause of the rigors. In the latter opinion I am, I think, in part borne out by the fact that, in those dying in the cold stage of ague, such congestion is found; and also by the observations of Bell and Shaw; for, when they irritated or simply pressed those twigs between the blades of the forceps, they observed that the muscles to which they proceeded were spasmodically affected.

As these and all other symptoms which manifest themselves during the cold stage of fever, may be traced directly or indirectly to impaired nervous energy, I shall, in the next Number, pass to the second stage, or that of excitement.

