# Errata

**Published:** 1828-04

**Authors:** 


ERRATA.

P. 185, 4th line from bottom, for *found* read *formed.*
— 191, 22d ditto ditto, after *cathartic* read *to.*
— 192, 8th ditto from top, after *nervous* read *influence.*

